# Supplementary material for: Mapping the global distribution and spread of the Plasmodium vivax-associated virus MaRNAV-1
Source: Virus Evol. 2026 May 23;12(1):veag031. doi: 10.1093/ve/veag031 (PMC13271372; doi:10.1093/ve/veag031)
Supplement: Supplementary_Materials_veag031 [file supplementary_materials_veag031.zip › SuppTable5.pdf]

Table S5 Primers used to detect MaRNAV-1 segments in primary human blood isolates.

Name	Sequence (5' – 3')	Application
Human RPS18 Fwd	ATGCAGAATCCACGCCAGTA	Human mRNA Detection (positive control)
Human RPS18 Rev	CCAGACCATTGGCTAGGACC	
MARNAV-1 Seg 1 Fwd	TGGCGGTCATTATGATCTCCA	MARNAV-1 detection
MARNAV-1 Seg 1 Rev	CGTGACACGAATAGGCCTCT	
MARNAV-1 Seg 2 Fwd	GCAGGGAGGAAGAAGCCTG	
MARNAV-1 Seg 2 Rev	GGCTTCGCCTCCTGAACTT	
