# Supplementary material for: Mapping the global distribution and spread of the Plasmodium vivax-associated virus MaRNAV-1
Source: Virus Evol. 2026 May 23;12(1):veag031. doi: 10.1093/ve/veag031 (PMC13271372; doi:10.1093/ve/veag031)
Supplement: Supplementary_Materials_veag031 [file supplementary_materials_veag031.zip › FigS1.pdf]

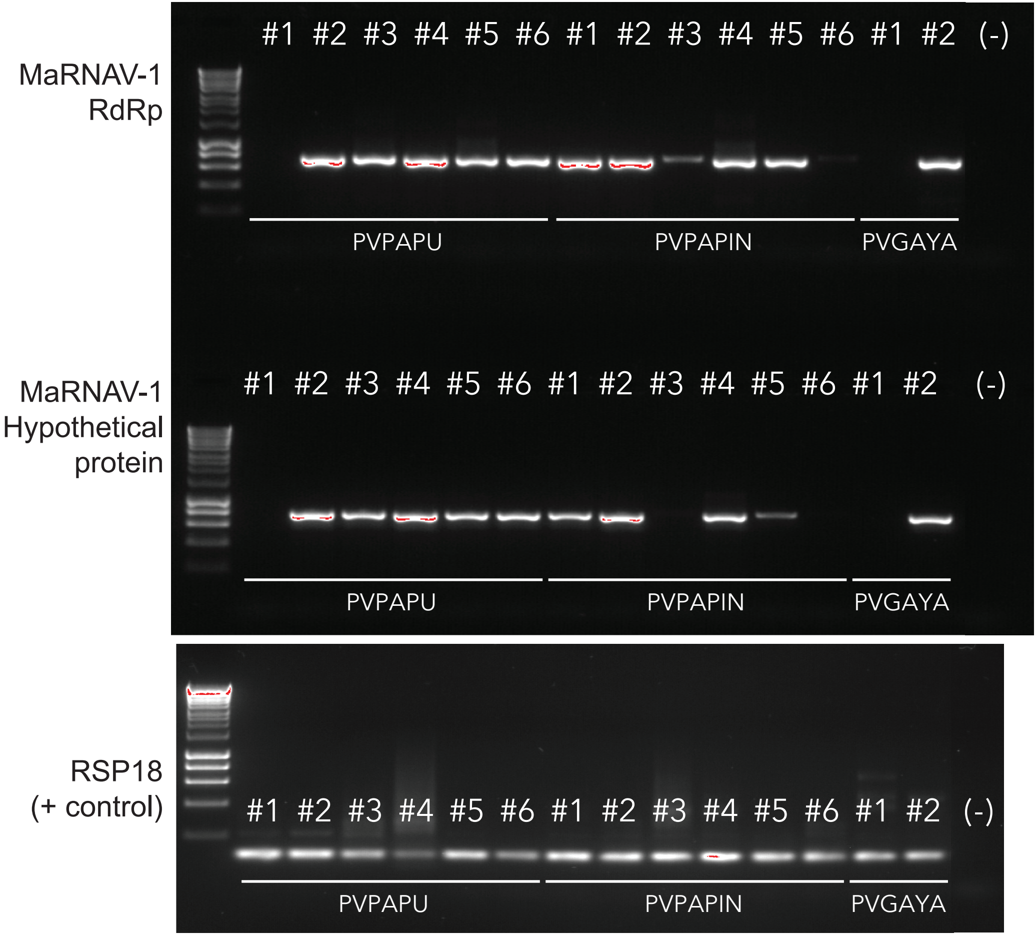

Figure S1 Visualisation of MaRNAV-1 segments in primary human blood samples with *P. vivax* infection that were pooled for sequencing. Abbreviations: PV = *P. vivax*; PAPU, PAPIN = Papua, Indonesia; GAYA = Gaya Island, Sabah, Malaysia
