# Supplementary material for: Mapping the global distribution and spread of the Plasmodium vivax-associated virus MaRNAV-1
Source: Virus Evol. 2026 May 23;12(1):veag031. doi: 10.1093/ve/veag031 (PMC13271372; doi:10.1093/ve/veag031)
Supplement: Supplementary_Materials_veag031 [file supplementary_materials_veag031.zip › FigS2.rev.pdf]

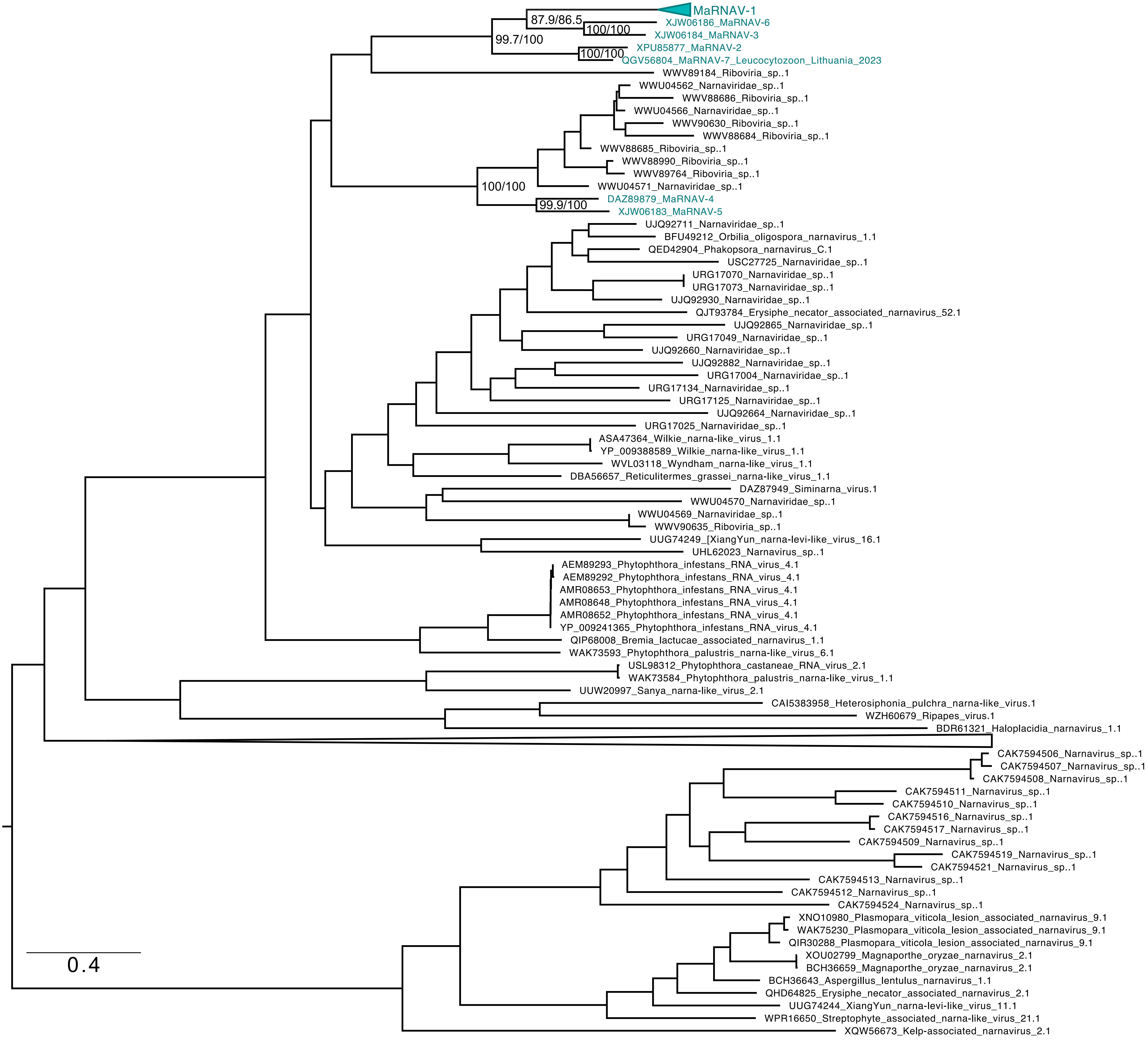

Figure S2 Relationship of known MaRNAVs within the *Narnaviridae*. Mid-point rooted maximum likelihood phylogeny scaled by the number of amino acid substitutions. MaRNAV lineages are denoted by green tips. Support values are shown at select nodes as sh-aLRT/UFBoot.
