# Supplementary material for: Mapping the global distribution and spread of the Plasmodium vivax-associated virus MaRNAV-1
Source: Virus Evol. 2026 May 23;12(1):veag031. doi: 10.1093/ve/veag031 (PMC13271372; doi:10.1093/ve/veag031)
Supplement: Supplementary_Materials_veag031 [file supplementary_materials_veag031.zip › FigS3.pdf]

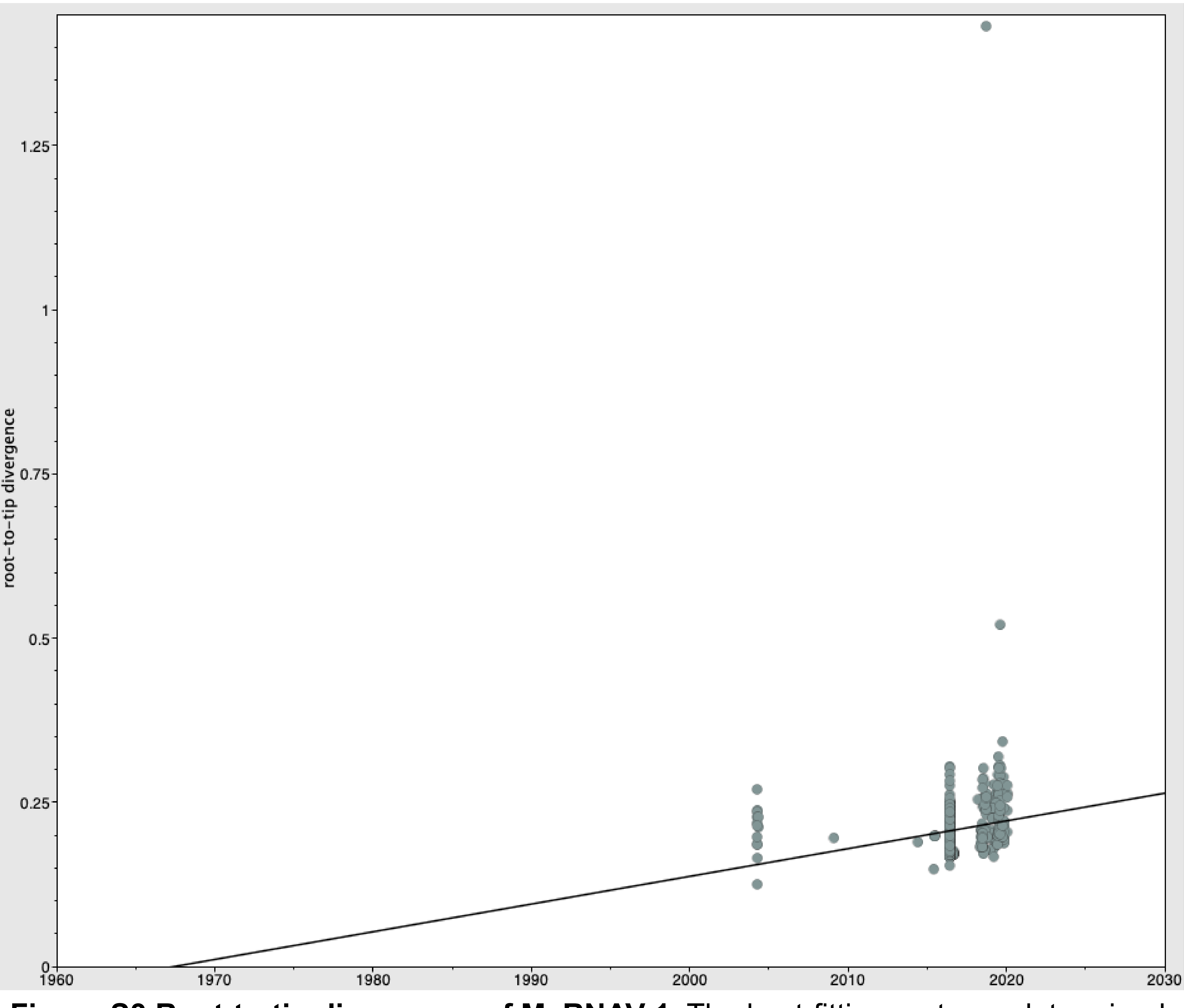

Figure S3 Root-to-tip divergence of MaRNAV-1. The best-fitting root was determined using the heuristic residual mean squared function. The results were visualised in TempEst. Year is shown on the Y axis..
